# The challenge of obtaining information necessary for multi-criteria decision analysis implementation: the case of physiotherapy services in Canada

**DOI:** 10.1186/1478-7547-11-11

**Published:** 2013-05-20

**Authors:** Francois Dionne, Craig Mitton, Tanya MacDonald, Carol Miller, Michael Brennan

**Affiliations:** 1 Centre for Clinical Epidemiology and Evaluation, Vancouver Coastal Health Research Institute, School of Population and Public Health, University of British Columbia, 7th Floor, 828 West 10th Avenue, Research Pavilion, Vancouver, BC V5Z 1M9, Canada; 2 Canadian Physiotherapy Association, Ottawa, ON, Canada

**Keywords:** Physiotherapy, MCDA, Resource Allocation, Priority Setting

## Abstract

**Background:**

As fiscal constraints dominate health policy discussions across Canada and globally, priority-setting exercises are becoming more common to guide the difficult choices that must be made. In this context, it becomes highly desirable to have accurate estimates of the value of specific health care interventions.

Economic evaluation is a well-accepted method to estimate the value of health care interventions. However, economic evaluation has significant limitations, which have lead to an increase in the use of Multi-Criteria Decision Analysis (MCDA). One key concern with MCDA is the availability of the information necessary for implementation. In the Fall 2011, the Canadian Physiotherapy Association embarked on a project aimed at providing a valuation of physiotherapy services that is both evidence-based and relevant to resource allocation decisions. The framework selected for this project was MCDA. We report on how we addressed the challenge of obtaining some of the information necessary for MCDA implementation.

**Methods:**

MCDA criteria were selected and areas of physiotherapy practices were identified. The building up of the necessary information base was a three step process. First, there was a literature review for each practice area, on each criterion. The next step was to conduct interviews with experts in each of the practice areas to critique the results of the literature review and to fill in gaps where there was no or insufficient literature. Finally, the results of the individual interviews were validated by a national committee to ensure consistency across all practice areas and that a national level perspective is applied.

**Results:**

Despite a lack of research evidence on many of the considerations relevant to the estimation of the value of physiotherapy services (the criteria), sufficient information was obtained to facilitate MCDA implementation at the local level.

**Conclusions:**

The results of this research project serve two purposes: 1) a method to obtain information necessary to implement MCDA is described, and 2) the results in terms of information on the benefits provided by each of the twelve areas of physiotherapy practice can be used by decision-makers as a starting point in the implementation of MCDA at the local level.

## Background

As fiscal constraints dominate health policy and planning discussions both across Canada and globally, priority-setting exercises are becoming more common to guide the difficult choices that must be made [1]. In this context, it is not only appropriate but also highly desirable to assess the value of specific health care services, as an assessment of value is necessary for priority setting on resource allocation either through the use of a threshold (minimum value per dollar spent) or through a formalized priority-setting process such as Program Budgeting and Marginal Analysis (PBMA).

A common approach to the assessment of the value of health care services is economic evaluation [2]. Economic evaluation is typically used in a threshold approach to resource allocation, meaning that interventions costing less than a threshold cost per unit of benefit are deemed worthy of funding. However, there are well-known challenges to the acceptability of economic evaluation as a tool to guide resource allocation decisions. A key such challenge is to “ensure alignment between the objectives assumed in economic analyses and the objectives facing decision-makers in reality” [3]. Specifically, economic evaluation as a priority setting tool assumes that the decision-maker’s objective is to maximize health gain [4] but we know that other objectives are also typically pursued [4,5]. One solution offered is that “the simple C/E ratio could be supplemented by information on other health effects for the patient, for example a descriptive account of expected improvements in quality of life; wider societal effects of the intervention, for example on the number of jobs created; and nonmonetary costs for the patient reported in natural units such as waiting time in days” [4]. Such a solution can in fact be formalized through the use of Multi-Criteria Decision Analysis (MCDA): “MCDA is aimed at supporting decision makers faced with evaluating alternatives, taking into account multiple, and often conflictive (sic), criteria” [6]. The criteria in MCDA are the ‘other health effects’, the ‘wider societal effects’ and the ‘nonmonetary costs’ referred to above, or put simply, the considerations that a decision-maker will typically take into account in making a decision on resource allocation. MCDA is typically used in formal priority setting processes such as Program Budgeting and Marginal Analysis (PBMA). Like economic evaluation, MCDA has methodological challenges, but in many contexts, because it formally includes most or all considerations relevant to decision-making, this approach, and the associated priority setting frameworks, fit the decision-maker’s perspective better [7,8]. One key methodological challenge of MCDA is the search for the necessary information. The necessary information is often not readily available for two main reasons. First, some of the criteria, while relevant to decision-makers, are typically not common research subjects. This would include criteria such as integration or access. Second, even when literature is available, the information must be contextualized before it can be used. This paper reports on an example of how this key challenge can be addressed.

In the Fall 2011, the Canadian Physiotherapy Association (CPA) embarked on a project aimed at providing a valuation of physiotherapy services that is both evidence-based and relevant to resource allocation decisions in health care organizations. This project originated more than one year earlier, in 2010, when the CPA Branch Presidents concluded that there was a need for more information on the value of physiotherapy services and asked the CPA national staff to consider developing a document that would address this need. After investigating the methodological alternatives, the CPA national staff decided to proceed with the MCDA framework.

In this paper we report on the methods used to obtain information necessary for MCDA implementation and provide a brief summary of the information that was produced. The objective is to show how a key challenge to the implementation of MCDA can be addressed and give a sample of the results. The full results which are in the final report are the starting point for an MCDA implementation. Actual examples of full implementation of MCDA within a priority setting process at the local level, building on the information produced in this project, are not included.

## Methods

MCDA involves the assessment of alternative actions on the basis of a common set of criteria [9,10]. The two key elements of the MCDA process are the alternatives to be considered and the criteria to be used. Possible alternatives are those options available to the decision-maker, for example changing the level of funding for a given physiotherapy service or program. The criteria represent the relevant considerations in assessing the impact of implementing any of the different alternatives. Criteria therefore depend on the decision-making context. Once possible alternatives have been evaluated on the basis of the selected criteria, they can be compared and recommendations can be formulated. The evaluation of each alternative provides an assessment of what would be lost, in cases of a reduction in funding, and what would be gained, in case on increases in funding. When contextualized, this valuation represents the marginal value of a service at the local level (as opposed to the total or average value) as the question that was posed with respect to each criterion was: what would be the impact on this criterion of an increase or a decrease to the current volume of service. The basic steps in MCDA are outlined in Table 1.

**Table 1:** MCDA steps

1	The **first step** is the development of relevant criteria. The criteria should be clearly defined and must relate to the overall purpose of the decision process. The objective in the development of criteria is to include all considerations relevant to the decision that has to be made and to provide sufficient clarity to ensure consistency in the translation of information about the alternatives into ratings.
2	The **second step** is the identification of the possible alternatives. In this case, the alternatives are the most common physiotherapy services. Each alternative [or in this case set of services] must be accompanied by the information required to assess it on the basis of the established criteria.
3	The **third step** is the formal evaluation of each possible alternative. This is done by rating each alternative on each criterion and calculating a composite score. Because the same criteria are used with all alternatives, the scores are comparable across all alternatives.
4	The **final step** is the formulation of recommendations. First, each composite score is validated to ensure that no process errors took place. Once that is done, each alternative can be ranked in relation to all others. Funding recommendations are then based on this ranking.

The first step in the application of MCDA to the valuation of physiotherapy services was to determine a set of criteria relevant to decision making on health care resource allocation involving such services. The perspective adopted for this project was that of a decision maker within a health region or health service delivery organization, as this was the primary target audience for this work. Based on previous priority setting work with Canadian health authorities and on the literature on priority setting [11-14] an initial set of criteria was proposed to the CPA and, through discussion, a final list of eleven criteria was developed (see Table 2). The criteria were defined in such a way as to ensure that overlap was minimized [i.e., they are meant to be mutually exclusive, as much as possible].

**Table 2:** Criteria and definitions

Resource impact	Impact on system-wide resource use
Quality of Life	This criterion deals with the absolute change in quality of life, i.e. a service that has a limited impact on quality of life could not rate to the top of the scale on this criterion.
Patient/ provider satisfaction	Deals with benefits of the service other than the direct impact on the underlying condition, for example, a service that is very personalized will rate higher here because, presumably, the provider would be able to allow to a greater extent for the client’s preferences [for example, regarding the nature of the activities, the location, the timing, the setting- group or alone].
Integration	This criterion is about the continuum of care [and goes beyond the health care system]. Does the service address a gap in the continuum of care that facilitates the clients’ transition from one program or service to another?
Access	This criterion measures the impact of the provision of a given service on the current utilization of other services, thereby possibly making these other services more accessible. For example, if a given service results in fewer hours per week of home care being required, then this service has freed up those hours for someone else to use. Some services will free up resources that way and some won’t.
Equity	Impact of the service on the health status of groups where there is an avoidable, unfair, and remediable health status gap.
Effectiveness	This is about the absolute effectiveness of the service. Just because a service is the best that can be done for an underlying condition does not mean that it is highly effective. Also effectiveness is measured with respect to the impact on the underlying condition itself or the impact on the consequences of the underlying condition.
Appropriateness	This criterion deals with the high level degree of match between a given service and the overall needs of the population, defined as the combination of the number of persons with the underlying condition and the impact of the underlying condition on quality of life. We should also consider here the availability of possible alternatives. Alternatives to be considered here can be privately provided services but also different services that are publicly funded. We are getting at the idea of the possibility of substitution with this criterion.
Acceptability	This deals with the relative ‘displeasure’ associated with the service delivery- amount of pain, discomfort
Implementation challenges	Risks associated with the implementation of given service change [for example, increased volume] but also degree of support- this would be measured, amongst other considerations, by the extent of public pressure in favour of a service.
Impact on future use of health care services [3+years]	This criterion is about the extent to which the provision of a physiotherapy service now is likely to affect the overall use of health care services down the road [at least three years from now].

Moving to step two, the CPA identified a set of service areas for assessment, based on relevant literature and similar briefings on value for money developed in the United Kingdom by the Chartered Society of Physiotherapy. The final list contained twelve service areas (see Table 3).

**Table 3:** Selected service areas for review

1.	Physiotherapy interventions for musculoskeletal conditions
2.	Physiotherapy interventions for low back pain
3.	Rehabilitation services in the intensive care unit
4.	Physiotherapy interventions for chronic disease management
5.	Rehabilitation services for chronic lung disease
6.	Rehabilitation services for cardiovascular disease
7.	Rehabilitation services following joint arthroplasty
8.	Rehabilitation services following stroke
9.	Physiotherapy services in the emergency department
10.	Home based rehabilitation services
11.	Rehabilitation services for falls
12.	Rehabilitation services for pediatrics

This research project was about the second part of step two which is to obtain information necessary to assess each alternative on the basis of each criterion. This was done in a three part process. First, a literature review was undertaken to identify peer reviewed papers that address the notion of value (as defined by the criteria). Search terms included the given service area along with ‘effectiveness’, ‘cost-effectiveness’, ‘value’ and a myriad of other terms relating to the identified criteria. Due to the breadth of the search, a systematic review was not attempted; rather key papers were identified and reviewed with the intent of providing insight into a given service area, as opposed to a comprehensive take on each area. Not surprisingly, for many of the criteria there was no, or very limited, research evidence. The second part of the search for information or evidence was a series of interviews with content experts for each of the twelve service areas. These content experts (n=1 to 3 depending on the service area) were identified by the CPA. Through one or more phone consultations, the literature review for each service area was critiqued and new information was generated where no, or insufficient, literature existed. This was an important part of the process as the literature only provided information on some of the criteria. The missing, but required, pieces of information thus came from expert opinion. It is in this combining of expert opinion with research finding that MCDA provides a pragmatic approach to valuation.

After drafting of an initial synthesis document by service area which combined the results of the literature review with expert opinion, there remained a need to ensure that 1) the information presented would be applicable at a national level (as opposed to the provincial or regional level) and 2) the assessments would be consistent across the service areas (noting that the content experts were only focusing on a single service area). For this purpose, in the third part of the process to acquire the required information, the CPA struck a validation committee comprised of eleven individuals from across Canada with a broad range of experience in physiotherapy. Over the course of 2 two-hour meetings, the synthesis document was reviewed in detail. In some cases the validation committee requested additional information from the literature and clarification of points made by the content experts. The synthesis document was then adjusted to reflect the comments from the validation committee, including additional research information and clarification of expert opinions, resulting in the final synthesis by service area. Steps three and four of the MCDA process were not included in this project as those steps are context-dependant by nature.

## Results: Key findings by service areas

In this section, we present the some of the key findings by service area. This section is limited to select key findings because full presentation is beyond the scope of this paper: we have 11 criteria and 12 service areas which means 132 cells of information which in the final report represented 55 pages of content. Where findings are based on published evidence, references are provided. When there is no reference, the findings are expert opinion, obtained as described above. Further details, including the key findings for each criteria, for each of the twelve service areas are presented in Table 4 (there again, where findings are from the literature, references are cited). Complete results can be found in the CPA report ‘Valuing Physiotherapy Services’ [15]. The findings presented here represent the minimum *starting point* required to implement MCDA at a local level. In the Discussion, we describe how these results can be used in an MCDA exercise in a health service organization.

**Table 4:** Key findings by criteria service areas

	**Resource impact**	**Quality of life**	**Patient/ provider satisfaction**	**Integration**	**Access**	**Equity**	**Effectiveness**	**Appropriateness**	**Acceptability**	**Implementation challenges**	**Impact on future use of health care services**
1. Physiotherapy interventions for musculoskeletal conditions	For non-urgent MSK patients, physiotherapists found to be highly effective gatekeepers to surgical care, providing appropriate assessment and management of patient needs; reduces costs of outpatient care [16]	Clear relationship between improved functioning and impact on quality of life	Patient satisfaction with physiotherapy treatment correlated to personal responsibility for managing disorder; recommend adjusting treatment to match attitude or attempt to change attitude [17]. As a provider, very rewarding area to work; client-centred approach; increases therapist’s drive to improve their skills	Physiotherapy can fill gaps for someone who is below threshold of MSK health; helps to raise client to minimum threshold so they can then move into the community and access personal trainers	Limited impact on concurrent use of other services: possibly better use of surgeons’ time	Disparity between patients not privately insured and those insured; similarly with on-site access versus off-site. Access tied to SES; few resources for those with low income	Outpatient multidisciplinary treatment program for sick-listed workers highly effective in improving physical functioning, physical disabilities, and kinesiophobia compared to usual care; no significant difference in cost- effectiveness on the societal level as compared to usual care	Orthopedic surgeons more likely to refer patients to PT than primary physicians; self-referral patients had lower PT visits than physician referred [17]. Need to increase therapy resources to address barriers to access [18]	Some services are quite uncomfortable (e.g., shoulders); but generally, clients do not stop due to discomfort; have to put treatment into broader picture of helping the client which may, at times, be painful	Public does not necessarily know what physiotherapy is; people who might benefit may not know how to access services or are unaware of how it would be beneficial. Need public and other professionals to be more aware of skills and impact of PT	Creating individualized programs and allowing for independent care outside of physiotherapy can result in lifelong changes: 8 weeks post-physiotherapy may not result in significant changes; however, large changes at 12-month; in addition, if re-injury occurs, costs are much lower
2. Physiotherapy interventions for low back pain	Physiotherapist-led pain management classes offer a cost-effective alternative to usual outpatient physiotherapy and are associated with less healthcare use [19]	Reduces pain and improves functioning, especially for chronic condition (confirmed through the administration of pre and post surveys)	Hands on individual care that results in patient satisfaction; individualized care with education is key element on satisfaction	Earlier position in the continuum of care would produce greater benefits; ironically, in rural areas, can typically get an MRI quicker then PT services	Main impact is on freeing up surgeon’s time by moving the triaging activity to the physiotherapist	No identifiable sub-population disproportionally affected by LBP although more women get treatment then men	Significant impact on risk of worsening disability and time off-work [20]. About 80 to 90% of all cases are resolved, i.e. patients experience a normal lifestyle except for the odd episodic recurrence	Incidence of LBP is steady but proportion of cases that evolve to chronic condition is increasing; this process accelerates access to treatment thereby reducing the risk of the condition becoming chronic.	Patients are more likely to participate in exercise programs that reflect their preferences, circumstances and abilities; recommend collecting patient preferences before starting treatment [21]	Requirements for triaging program: Cooperation from surgeons; Specialized training for the physiotherapist	Long-term impact will be on the proportion of cases that become chronic (chronic LBP affects mobility which has psychological impacts as well as physical impacts through the limitation on the ability to exercise)
3. Rehabilitation services in the intensive care unit	With physiotherapy, functional ability at time of discharge from ICU is higher, leading to reduced costs such as multi-system de-conditioning with long- term bed rest	Impact of ICU physiotherapy on QoL is mainly through prevention of problems resulting from an ICU stay. These problems are a direct determinant of where patients goes next, e.g., nursing home or own home	Significant provider satisfaction in this field in assisting people to move earlier along with greater patient connection; physiotherapy is a constant; promotes relationship building	ICU is extremely multi-disciplinary; no practitioner can act in isolation and therefore coordination occurs across disciplines, in this context, physiotherapist chart notes have a direct impact on how the patient is treated on the ward	PT can affect LOS in ICU	ICU population is heterogeneous; equity not an issue	Two key areas of impact: Early mobility Ventilator weaning	Patients are becoming far more complex with co-morbidities – physiotherapists look at patients holistically versus possible fragmentation of specialized services	Involves hard work but no different than other PT services	Specialized equipment required	Ability to go home earlier with physiotherapy service ; however, longer term utilization is less likely to be impacted
4. Physiotherapy interventions for chronic disease management	Service is found to be sufficiently cost-effective to be included in the coverage provided by some privately-funded extended health care plans	Because of the mobility concern, the impact of physiotherapy on QoL is connected primarily to increased level of activity and functioning. Many disease specific research findings	Ranges of improvement but chronic disease by definition will not be ‘curative’; PT best viewed as an integral part of multi-modal team of care	Without physiotherapy, patients would be on waitlists for physician services or surgery; assists with filling gaps	When physiotherapy conducted alongside physicians, physicians’ capacity increases	No impact	Because patients’ problems are multi-faceted and require multiple interventions (e.g. medication, surgery), PT role in designing exercise programs that take all of these factors into consideration is central to overall effectiveness	Growing problem, especially with an aging population	Important to measure and track progress as an incentive	Expertise is available, especially if physiotherapists are used to plan and supervise activities, while assistants provide instruction and oversee individual exercise programs (see: CLCS model in community centres in Quebec)	Significant prevention potential that can have a large impact on future use of resources
5. Rehabilitation services for chronic lung disease	Multidisciplinary, outpatient pulmonary rehab (PR) program substantially reduced health resources use in patients with moderate, severe and very severe COPD. The mean incremental cost of adding rehabilitation to standard care was a savings of $152 per patient [22]	PR shown to improve quality of life (Rubi; McCarroll); PR deals with physical function, but also with the psychological aspects through education	Patients who have received PR often want to be re-admitted after their next exacerbation	There is poor continuum of care for COPD patients. Current care is focused on responding to exacerbations	Use of PR results in less exacerbations, fewer ER visits and reduced number of unscheduled GP visits	COPD does not disproportionally affect any specific ‘disadvantaged’ group	There is strong evidence demonstrating a reduction in dyspnea, increased exercise tolerance, improved health related quality of life and cost-effectiveness [23]	COPD is a significant chronic disease in terms of incidence and prevalence: fourth or fifth leading cause of death	Patients typically want to return to treatment	No specialized resources needed; physiotherapists can be trained quickly in the specifics of this service; exercise equipment used is standard	Patients receiving PR are, in the long run, more likely to stay at home longer, therefore postponing institutionalization
6. Rehabilitation services for cardiovascular disease	Outpatient CR less expensive than inpatient yet similar effectiveness	CR significantly improved QoL scores, reduced depression and had positive effect on psychosocial measures	Service can be fully tailored to the client’s situation	Clients come from diagnosis of cardiovascular condition then transition to local, ongoing, community services; CR plays an essential role in facilitating this transition	Impact on the use of other health services is not immediate (except for length of hospital stay)	Women, the elderly, ethnic minority groups access CR less. Very little information on why subgroups have lower rates of access	CR reduces the risk of cardiac and general mortality rates by 25-30%	There is a growing referral rate AND a growing uptake rate because of increased awareness (referral rate) and improvements in services (uptake rate)	The services are mostly about teaching so there is no physical pain. Changes in lifestyle being promoted can be difficult to adopt	None noted	Services reduce the likelihood of recurrence of the problems and reduces the seriousness of future problems
7. Rehabilitation services following joint arthroplasty	When comparing the cost-effectiveness of an accelerated perioperative care and rehabilitation protocol with that of a more standard protocol for patients treated with total hip arthroplasty, beginning from the first visit before the operation to one year postoperatively, a study found the accelerated intervention to be more effective with an average of $4000 reduction in treatment costs with a 0.08 QALY gain; also more cost-effective for total knee arthroplasty with no difference in QALYs [24]	PT provides both earlier functionality and a better end point	Postoperative, active physical therapy increases satisfaction and helps to meet patient expectations [25]	Impact on continuum of care comes from accelerating patient’s progression through the care process	Will reduce doctor visits	More difficult to access PT services in rural settings	Using team approach, patients had large improvements in outcome measures during the rehabilitation stay and 6-month follow-up [26]	Joint arthroplasty volume is driven by demographics	High acceptability	No significant HR or equipment challenges	No evidence of impact on future use of health care services (3+years)
8. Rehabilitation services following stroke	Very early mobilisation (VEM) more cost-effective than standard care and improved outcomes	Research findings still lacking; recent innovations in diagnosis, management, and rehabilitation have resulted in measurable improvements in clinical and functional outcomes after acute stroke; however, despite improvements in medical management, quality of life is not necessarily improving post stroke [27]	Programs are meant to be patient-centered: this is the goal; limitation is in resource constraints which reduces ability to customize treatment plans	Key component of the continuum of care; If there is not sufficient physiotherapy services LOS is longer and/or the patient does not do as well	Very limited impact on the concurrent utilization of other services	Increased odds of problems from a past stroke associated with failure to access OT/PT services, lower monthly income, and age	Comparing specialized outpatient therapy to no treatment, 14 RCTs found that therapy-based outpatient rehab was associated with a reduction in the odds of poor outcome and increased daily living and personal activity scores	Stroke is a significant condition in terms of incidence; physiotherapy is an integral part of its treatment	Stroke causes fear in patients, which increases treatment acceptance rate; physiotherapy focuses on restoring physical function and in so doing, provides positive feedback	Requires more rehab beds and/or specialized units	Improved physical function and has direct impact on social function; minimizes the future use of health care services
9. Physiotherapy services in the emergency department	Can reduce LOS for some patients; facilitates flow in the ER	Services address fear and uncertainty around risks when discharged	Potentially better client satisfaction: less pain, reduces short-term disability, improves function and safety	Important ‘triaging’ role in the continuum	Sizeable impact on rate of return visits to emergency	Rate of emergency visits not clearly related to being part of any disadvantaged populations	At system and provider levels, there is limited research evidence on the value of an emergency department physiotherapy service; at patient level, there is high-level evidence of benefits in terms of improved pain control and reduced disability in the short term	There is an increase in ED attendances, therefore an increased need for emergency PT services	Sometimes ‘forces’ the realization that the patient is at a time of life where there is a loss of independence and a need for mobility aids or assistance	Increased volume comes with a need for observation beds and sub-acute beds	Patients are flagged earlier for present and potential problems and can be followed/assisted in the community
10. Home based rehabilitation services	Significant cost aversion; mobility assessment, keeping people independent in their homes; prevention of falls and providing a safe environment within the home context	Impact of PT can include increased social interaction; improved personal and domestic activities; improved health status; improved subjective quality of life; reduced caregiver burden	Patient satisfaction is clear (but typically is not tracked by formal instruments); one measure of satisfaction is that the clients pay for subsequent visits; verbal feedback from clients is very positive; while anecdotal, the high level of satisfaction is clear	Service is extremely relevant to service integration; big gap in the continuum of care from hospital to home; a lot of people discharged from the hospital and in need of home-based service but are not receiving it or receive it in a very limited manner, i.e., no active rehab post discharge, rather patients are given a walker or basic level of information	Reduces LOS and hospitalisations	Inequities exist between Provinces: those without financial means do not have access to home-based rehab services in some Provinces; those with chronic conditions are more vulnerable and need more follow-up; currently, there is no support from the public system to help these individuals	When comparing adults 70 years or older with one or more functional problems who received a home-based programme of occupational therapy and physiotherapy to a control group, a significant reduction in mortality rate was found (5.6% vs 13.2%); individuals with a moderate risk of mortality in the intervention groups also showed a significant reduction at 16.7% vs. 28.3% [28]	Home-based therapy increases access, in particular for patients with greater medical complexities	Main issue is the payment required for services	Have to have the right provider: not every physiotherapist can provide this service; broad experience base is required to be effective and proficient; therapist works on their own which means there are no second opinions; some anxiety in providing in-home services and worker safety can be a concern	Home-based services are expensive with respect to time to travel and low volume however this needs to be considered in light of potential decrease in utilization of future service needs; in the long term, this is a very efficient use of societal resources
11. Rehabilitation services for falls	Treatment for falls was 1.8 times more costly than implementing a fall prevention program	Specialized balance program for women with osteoporosis significantly improved quality of life, physical function, symptoms, social interaction and overall wellbeing [29]	Falls prevention programming is a new field, to date has not drawn adequate attention	Not really part of a continuum of care in most cases	Fall prevention service does not reduce client’s use of other services; greatest impact on future service use	Programs tend to target seniors and diabetics	Exercise program significantly reduces the risk of death, of falling and hospitalisation or transfer to a nursing home	Need to get out in front to provide prospective services instead of providing service retrospectively	No physical risks or discomfort but psychological ‘discomfort’ as fall prevention associated with a loss of independence	More awareness with health care professionals generally	Substantial impact especially in the subset of cases where falls can be avoided
12. Rehabilitation services for pediatrics	Getting right programs in place early can make a lifelong difference in health outcomes and lead to very significant savings	Movement is freedom; for children who have difficulty getting involved in activities, these services open opportunity for participation	Physiotherapist is the health care professional in closest contact with the patient and his/her family; relationship that develops is potentially unlike any other health care profession; very personal in nature; physiotherapists best understand the child’s disability and so can relate very well; becomes very strong advocates for the patient and family	The service definitely addresses a gap; if this service was not in place, by the time the child reached adulthood they would be so far behind in their development they could never catch up	Some surgery avoidance; some reduction in GP visits	Many disadvantaged groups do not typically go to the hospital for services; if rehab services are in the community and/or school or community centre, access to health care is more likely: practitioners will often see individuals who have not accessed any other service in the system	Many studies have shown effectiveness; studies are typically small, but results are consistent across conditions	Children do not respond as well in adult facilities	Typically, very well received	Baseline services are not a challenge - new grads can do this	Early intervention has significant impact on reducing future utilization of services, including prevention of secondary surgeries

### Pediatrics

The cost of providing pediatric physiotherapy services tends to be higher than treatment for adults, however the long-term benefits and decreased burden on future use of care services can be significant. Besides the expected direct impact, for example, the direct impact on children with juvenile idiopathic arthritis [30], with cerebral palsy [31], or with cystic fibrosis [32], there are two important benefits of pediatric physiotherapy that emerged: 1) the physiotherapist typically develops a supportive relationship with both the child and his or her family. In this role, the therapist is an essential source of information and education making the physiotherapist a valued link to, and guide through, an often-times overwhelming care process for children and their parents; and 2) pediatric physiotherapy services play an important role in the transition to adulthood. Therapists can act as a bridge between programs to ensure the continuation of treatment while transitioning from child to adult care.

### Home-based services

Home-based physiotherapy services are highly effective for many health conditions, including frailty in elderly adults [33], ankle fractures [34], stroke [35], heart failure [36], breast cancer [37], and recovery from hip replacement surgery [38]. For such conditions, home based interventions have been shown to lower mortality rates related to falls [28,39] and the risk and rate of falls in older adults [40], reduce the number of nursing home admissions and hospitalisations, and decrease hospital length of stay.

Home-based physiotherapy programs are critical to service integration, providing a much-needed link between hospital and home. Home-based physiotherapy services can also help with a social issue: social isolation is often an issue for older clients and clients with more complex conditions; with physiotherapists providing in-home care, patients receive regular visits and consistent monitoring and follow-up.

### Intensive Care Units (ICUs)

The most common use of physiotherapy in ICU is to improve function for patients on mechanical ventilation [41]. Improving function has been shown to reduce dependency and promote earlier weaning, which in turn decreases hospital length of stay and increases quality of life [42-44]. With a reduction in hospital length of stay, along with increased function and fewer patient complications, physiotherapy treatment is highly cost-effective, reducing both the burden on acute care services and future health care service use [45,46]. Further, because treatment prevents critical weakness and increases functional ability [45,47,48] patients are less likely to be discharged to a care facility and are more likely to return to their home.

### Cardiovascular rehabilitation

Cardiac rehabilitation services support patients when transitioning from hospital to the community by helping with linkages to services within the community. This helps to ensure that client care continues after discharge. Such linkages also help to promote social engagement, adoption of healthy behaviors and provide support for self-managed care. Along with a resulting reduction in hospitalisation rates [49] and improvements to physical activity, smoking cessation rates, systolic blood pressure, weight loss and total cholesterol [50,51], cardiovascular physiotherapy services also provide a means of enhancing the surveillance of higher risk patients while providing personalized, tailored care that leads to improved psychosocial function.

### Emergency

Physiotherapists in emergency departments can improve pain control [52] and reduce short-term disability [53]. Early access to physiotherapy for this purpose can impact current and future use of health care services. Physiotherapists also aid in discharge planning by providing community program information and recommendations for mobility aids. Such assistance facilitates the continuation of care which in turn can alleviate patients’ fear of the acute event reoccurring while supporting a safe return to the home and community.

Emergency department physiotherapy programs can also decrease hospital length of stay and wait-times, in particular for minor musculoskeletal injuries [54]. Further since emergency departments are often a patient’s first point of care, clients who would benefit from physiotherapy interventions can be flagged early on in the care process directly impacting current and future use of health care services.

### Stroke

Research shows that physiotherapy services for stroke patients aid in the prevention of subsequent acute events while supporting a patient’s ability to live independently [55,56]. Physiotherapy services were also found to be a key component in the continuum of care, supporting patients in their transition from hospital to home [35]. This is particularly true when treatment is provided early and through a specialized stroke unit [56-58], with a dose-dependent effect being present [51]. High intensity physiotherapy programs, task-specific therapies and individual discharge planning all contribute to improved outcomes.

Outpatient physiotherapy programs for stroke patients are also effective. It was found that when outpatient rehabilitation programs were reduced, the length of stay in hospital increased along with rehospitalisation rates and overall costs [59,60].

### Musculoskeletal conditions (MSK)

With programs focusing on client self-management and independence, physiotherapy services are highly valued as an effective tool in the promotion of injury recovery and prevention of acute events [61]. Furthermore, there is a clear, positive relationship between increased physical functioning and improved quality of life.

While the initial costs of physiotherapists treating MSK patients are higher because of the requirement for experienced therapists, patients tend to require fewer visits over time. Care costs can be further reduced by using physiotherapists in triaging of patients: experienced physiotherapists can act as gatekeepers to surgical care, providing appropriate assessment and management of the patient’s condition [62-64].

### Low back pain

Physiotherapy for patients with low back pain is highly effective in reducing both acute and chronic pain while significantly limiting the risk of increased disability and chronic conditions [65-67]. Research suggests that between 80 to 90 percent of all lower back cases can be resolved through participation in rehabilitation programs. Rehabilitation programs are also cost-effective [68-71]. Prompt access to a dedicated physiotherapist for new cases of low back pain, in particular for high-risk patients, often pays for itself by reducing the burden on other health care services and promoting self-managed care. Brief, simple and early interventions that include providing information, reassurance and encouragement to engage in regular physical activity have resulted in economic gains measured one year after patients received the intervention, with no long-term negative effects [71].

Physiotherapists can also assist in the triaging of patients to ensure that only those requiring an MRI and a surgeon consult receive a referral for such. Acting as a gatekeeper to surgical care, physiotherapists are able to reduce patient treatment costs and significantly impact surgical wait-times.

### Joint arthroplasty

Overall, effectiveness studies indicate that patients who underwent joint arthroplasty *and* participated in physiotherapy programs experienced improved outcomes [72] with the greatest health gains achieved from early intervention such as starting rehabilitation 24-hours post-surgery [73]. Benefits included a reduction in pain and an increase in joint motion range, strength and balance [73,74]; short-term functional milestones were also attained within a shorter timeframe [24,75]. Early intervention had a positive impact on the length of hospital stays resulting in programs that are highly cost-effective [24,76,77]. Overall, inclusion of physiotherapy services in the care continuum had a significant impact on treatment costs [78]. Discharging patients direct to home with supportive therapy was also found to be more cost-effective than remaining in hospital with no difference found in health outcomes.

### Chronic diseases

There is strong support for the use of physiotherapy in the prevention and treatment of chronic diseases, including hypertension, emphysema, type II diabetes and obesity [79-84]. Studies have shown that patients who participated in individualized exercise programs had fewer emergency readmissions and physician visits and greater quality of life than patients in usual care. Physiotherapy programs also facilitate participation in community programs that enhance and maintain physical wellbeing, and this in turn can significantly impact future use of health care services. Physiotherapy is an integral part of the inter-professional team in the management of patients with chronic diseases.

### Falls

Physiotherapy is a highly effective tool in the prevention of falls and fall-related injuries both in hospital [85], as well as in the community [86-88]. In the community, the effectiveness of physiotherapy programs is significant with services improving the strength, motor function and balance in older adults who had previously experienced a fall event [89]. These effects contribute to reduced mortality rates, rates of hospitalisation and transfers to a nursing home allowing individuals to live independently in their homes. Similarly, the implementation of a falls-prevention program in an orthopaedic hospital can result in a significant decrease in fall incidence [85], fall-related morbidity and service costs.

Quality of life measures indicate that participation in a falls-prevention program improves a patient’s confidence and reduces the fear of falling that often restricts overall physical activity [90].

### Chronic lung disease

There is strong evidence to support the effectiveness of pulmonary rehabilitation services for patients with chronic lung disease, with program participation correlated with decreased rates of dyspnea, exacerbations, and emergency room and physician visits [23,91,92]. Physiotherapy services were found to be cost-effective [93-98] and in some cases a program’s net cost was negative (i.e. the program produced net savings): for patients participating in outpatient pulmonary rehab programs, evidence suggests that patient total health resource use is lower compared to usual care. Rehabilitation programs also decreased medication use, the number of ICU admissions over time, and assisted patients in managing their condition, enabling them to remain in their homes longer [99].

## Discussion

In the context of choices that must be made because not all activities can be carried on as they were due to financial restrictions, information about the value of any given intervention is very useful [100]. A common framework for generating such information is economic evaluation where the cost per Quality-Adjusted-Life-Year (QALY) gained through a given intervention is estimated. The estimated cost per QALY gained however only addresses the impact on the life expectancy and on the quality of life of the clients or patients. In making decisions about allocating limited funding, decision-makers typically consider other objectives in addition to the direct health impact, with equity and access, for example, being often cited [101]. Moreover, economic evaluation is focused on specific end-points which are typically directly related to the condition, or potential condition, being addressed, for example, the extent to which physiotherapy services would impact a specific measurement of the progress of juvenile idiopathic arthritis. Because of these limitations, when the CPA decided to address what they felt was a gap in the available information on the value of physiotherapy services, it made the decision to address this gap through the application of MCDA. The overarching thinking behind this decision was that, as healthcare organizations face increasingly tougher choices, the limitations of QALYs as a resource allocation tool will push organizations toward more formal resource allocation frameworks that use MCDA in their evaluation of alternatives and physiotherapy services will be more likely to receive fair consideration if the health care organizations have access to accurate information. PBMA would be one of these frameworks. The choice of MCDA was not primarily guided by the relative level of difficulty in implementing a QALY approach versus MCDA. There was what was perceived as a shortcoming in information on the value of physiotherapy services and it was decided to put effort in the MCDA approach provided a greater potential for impact.

The result was a comprehensive report summarizing the value of each of twelve areas of physiotherapy services with respect to each of eleven criteria that were thought to represent all relevant considerations in making decisions about funding involving those services. Some key findings in terms of benefits of physiotherapy services are presented in this paper. It must be recognized that this paper is not reporting on an implementation of MCDA, or of a prioritization exercise. In fact, what the CPA has done is supply health care organizations in Canada and elsewhere with a base of research work necessary for the implementation of MCDA, as part of a resource allocation framework such as PBMA. The findings can be used as a starting point within any local MCDA implementation. It is not the role of the CPA to contextualize the information, assign weights to the criteria, or even suggest that only the criteria listed here should be used, or to actually rate the impact of service volume changes. These steps are the responsibility of local health care organizations. An organization that decides to implement the MCDA framework to guide resource allocation would have to: 1) determine locally relevant criteria and weight them (these could be different than the criteria used in this study but it is not expected that there would be significant differences); 2) identify possible service volume change options that make sense in their context (which depends on the existing mix and volume of services provided); 3) assess the impact of each option on the basis of the selected criteria (this is where the information contained in the CPA report comes into play and provides a necessary starting point, i.e. necessary but not sufficient information). Note that the breakdown of areas of practice may not perfectly fit a given local context, in which case, the relevant areas from the twelve used here can be combined; and 4) rank the options and make decisions. All these steps are standard practice in most prioritization framework, and are part of the PBMA, for example.

Our objective here was not to provide one more case of PBMA implementation but to address a common criticism of PBMA or any other process that includes MCDA: that the required information is either not available or too difficult to obtain making such processes unimplementable and therefore only theoretical constructs. . In terms of information generation, the literature review posed no unusual challenge. As was expected, in the grid of criteria by service area, many of the cells were left blank after the review. The recruitment of experts was done by the CPA and didn’t seem very difficult for two reasons: this was a project of the CPA and many members are very supportive of their organization and it is a project that many members can relate to and specifically support. Furthermore, the demands are not overly burdensome as each expert was asked to participate in one or two calls of one to two hour each. What is more challenging is explaining to the experts what is needed from them which is to provide a response to the best of their knowledge and not limit their answers to what they know is research evidence- we really wanted their expert opinion. While this did not come naturally to some of the experts involved, all ended up contributing as was needed. Putting together a validation committee was no more challenging than recruiting experts for the same reasons. And just as was the case with the experts, it is necessary to have a full explanation of the process and some basic training in MCDA before the committee can start to work. The main challenge with validating the local data to the national level was understanding how much of the expert opinions were shaped by unique local circumstances. This was addressed by first identifying where this might be the case, going back to the local expert for further information, and then reconvening the validation committee. The key lessons from this experience were: 1) there has to be experts that buy into what is being done, reluctant participation would defeat the process; 2) explanation of the process and its goals and basic training is necessary before the experts can be asked questions. Finally, it must always be remembered that the ultimate goal is to obtain the best existing information, sometimes experts feel uncomfortable with expressing their opinion in response to a question but if it is the only available information then it becomes the best existing information. In our project, we found some initial hesitation in some cases but all experts were able to overcome it. The main limitation of this paper and the supporting report is the extent of the resources available for this project. For many criteria, the principal source of evidence was expert opinion and this was provided on a strictly voluntary basis. There was sufficient input into the process to produce validated results but, without a doubt, more resources would have produced a more refined report. However, a benefit of the MCDA approach is the transparent nature of the process which allows ongoing updating of the results. As new studies are published or as more experts can devote time to this analysis, findings can be continually updated, by area of service or by criterion. And further areas of service can be added.

## Conclusion

As the growth in public health care funding slows, more difficult choices about what to fund and what not to fund must be made. In this context, relevant and accurate information about the marginal value of any health care interventions is essential for proper resource management. MCDA can be a very effective means of producing such valuations which can then be used in whatever priority setting process is implemented. However MCDA requires evidence on aspects of value where there is typically very little research evidence available. In this paper we have described an approach to addressing this challenge. The results presented are valuable for two reasons. First, a pragmatic approach to the generation of necessary evidence is presented. While this approach may seem rather obvious, the fact is MCDA and priority-setting processes that employ MCDA are often denigrated on the basis of the implied demands for information and the challenges that this poses. Second, this paper also provides a glimpse of the findings that were generated which may lead some readers to refer to the final report as a solid starting point for an application of MCDA involving any of the twelve areas of physiotherapy services studied.

## Competing interests

This project was funded by the Canadian Physiotherapy Association. The views expressed in this paper are those of the authors and not necessarily those of the Canadian Physiotherapy Association.

## Authors’ contributions

FD and CM have made substantial contributions to the conception and design of the study, to the acquisition of data, and the analysis and interpretation of data. They have drafted the manuscript. TMD and CM have made substantial contributions to the conception and design of the study, to the acquisition of data, and to revising the manuscript critically for important intellectual content. MB has been involved in revising the manuscript critically for important intellectual content.
